# Delivery of Extra-uterine Fœtus through the Abdominal Walls after Four Years

**Published:** 1871-07

**Authors:** J. H. Beech

**Affiliations:** Coldwater, Michigan


Article IV.—*Delivery of Extra-uterine Fœtus through the Abdominal Walls after four Years.* Reported by J. H. Beech, M.D., of Coldwater, Michigan.

Mrs. Alexander Jameson came to my office August 12, 1870. She is 28 years of age—living with second husband—a well proportioned woman, weighing about 98 pounds—florid complexion, auburn hair, sanguine temperament—had one daughter, by former husband, now eight years old.

Menses always normal until about four years ago, when she believed herself to be pregnant. About eight weeks after regular menstruation she had a "miscarriage." Attended by her family physician, Dr. R. Bevier, of Plymouth, Ohio, where she then resided. Eight weeks from above accident she thought she felt "quickening," the abdomen and mammæ gradually increasing. From that time she "felt motion," until the period at which she expected labor, and to which time her size increased in normal degree. Some difficulty existed with the urinary organs, which she supposed grew out of her enciente condition. In other respects she was healthy. Apparent labor came on at the expected time, and the prospect of delivery was not given up for several days. After this period, the size of the abdomen and mammæ diminished, and with the subsidence the abdominal tumor became harder and more movable.

Menstruation returned after a time. She now suffered occasional lancinating pains in the tumor, which had seemed to become more prominent of late. It could now be moved as freely as a short pedicled, ovarian tumor can ordinarily be. It filled the left side of the abdomen a very little more than the right; was not very sensitive, and was even and uniform to external touch and sight. Per vaginam: uterus normal, very slightly inclined toward right of pelvis, and could be moved independent of tumor.

Dr. Frank Buckland was called in consultation. Her opinion that it originated in conception was presumed to be correct; but as the outlines of a fœtus could not be made out, it was believed to be mainly fibrous growth. Its removal being advised, board in the city was engaged, and the time set for the operation in about three weeks, which would be at close of next menstruation. At the appointed time I received a letter from the husband, stating that Mrs. J. was ill of "chills and fever."

More than two months after, i. e., about the middle of November, Dr. J. L. Hagerty, of Jamestown, Ind., informed me that he had been attending Mrs. J. through a very severe fever of typho-malarial character, but which exhibited such eccentricities that Dr. T. McNabb, of Fremont, Ind., had been associated with him in charge of the case. She was now slowly convalescing, and anxious to have an operation before she should get strong enough to ride to Coldwater.

Tuesday, November 22, 1870, visited Mrs. Jameson for operation, Drs. S. S. Cutter and Frank Buckland, of Coldwater, Mich., Drs. J. L. Hagerty, of Jamestown, Ind., T. McNabb, of Fremont, Ind., and H. D. Wood, of Angola, Ind., assisting. Patient feeble, but resolute; abdominal walls drawn tightly over resisting mass, which presented but one elastic point, that was about three inches from umbilicus, on a line towards anterior superior spinous process of left ilium.

The patient and friends desired, and received, a promise that if unusual danger presented, the operation should cease, and whatever wound had been made be closed.

Anæsthesia having been produced by chloroform, an incision in linea alba was made about eight inches long. The omentum majus was found adherent to the anterior wall of the abdomen, and by its posterior surface to the entire front of the tumor. Avoiding the conspicuous bloodvessels, an incision was made through the anterior portion of the omentum and the abdominal walls pushed back of the mass, which could not be raised in the least degree from the pelvis. A trocar and canula were plunged into the elastic portion, above mentioned, and about f℥ij of thick, grumous, purulent fluid were evacuated. Evidence of dangerous prostration appearing, as sudden sinking of the pulse, gasping respirations, extreme pallor and shriveled surface, it was unanimously agreed that the abdomen should be closed without further operative efforts; Drs. Cutter and McNabb giving their entire attention to efforts at resuscitation of the p atient. Drs. Wood, Hagerty and Buckland rendered most efficient and expeditious service in bringing forward the abdominal walls, and the wound was made secure with interrupted sutures and adhesive straps. Scarcely any blood had been lost, or serum discharged.

Dr. Hagerty subsequently informed me that the prostration continued in extreme degrees for fourteen hours. Pulse at times could not be counted; vomiting and retching severe, followed by eructation for several hours.

The pulse came to 135, when irregular chills came on, but subsided under the use of narcotics, tonics, preparations of potassa, and local detergents and antiseptics. Suture pins were removed on the fourth, fifth and sixth days.

A considerable portion of the wound healed by first intention; but a space opposite the puncture made with the trocar remained open.

Dr. Hagerty leaving for Chicago, some two weeks after the operation, the patient was placed in the care of Dr. T. McNabb, of Fremont, Ind., who pursued the same general course, and had the satisfaction of establishing permanent convalescence.

Dr. McNabb leaving for Ann Arbor, some three weeks after the time when he took charge of the patient, she came under the care of Dr. H. D. Wood, of Angola, Ind., who continued the same general course of treatment for some time.

The communication between the track of the trocar and the unclosed portion of the external wound becoming more direct and patulous, Dr. Wood was enabled to reach with a probe crepitating surface, which he correctly diagnosed as fœtal bones, and on the 24th day of January, 1871, assisted by Dr. I. K. Dunning, of Angola, Ind., (under local anæsthesia by æther spray), enlarged the opening, and removed a partially decomposed fœtus, weighing about seven pounds, of male sex.

I have delayed the report of this interesting case, for an answer to a communication to Dr. Wood, asking for details of the final operation, but have received none. From the husband and from Dr. Hagerty, who, on his return, resumed charge of the patient, I have learned the essential facts, and that convalescence has been steady. She menstruated April 20th; some menstrual flow passing through the external wound. Menstruated again at regular period, showing no color or unusual discharge through the wound. She is now attending to some household duties, although there is still a slight discharge from the external wound, which is about three lines in diameter, and when injected will hold about f℥ij.

